# Artificial intelligence to estimate the tear film breakup time and diagnose dry eye disease

**DOI:** 10.1038/s41598-023-33021-5

**Published:** 2023-04-10

**Authors:** Eisuke Shimizu, Toshiki Ishikawa, Makoto Tanji, Naomichi Agata, Shintaro Nakayama, Yo Nakahara, Ryota Yokoiwa, Shinri Sato, Akiko Hanyuda, Yoko Ogawa, Masatoshi Hirayama, Kazuo Tsubota, Yasunori Sato, Jun Shimazaki, Kazuno Negishi

**Affiliations:** 1 Department of Ophthalmology, Keio University School of Medicine, 35 Shinanomachi, Shinjuku-ku, Tokyo, 160-8582 Japan; 2 OUI Inc., DF Building 510, 2-2-8 Minami-Aoyama, Minato-ku, Tokyo, 107-0062 Japan; 3 Yokohama Keiai Eye Clinic, Courtley House 2F, 1-11-17 Wada, Hodogaya-ku, Kanagawa, 240-0065 Japan; 4 Department of Preventive Medicine and Public Health, School of Medicine, Keio University School of Medicine, 35 Shinanomachi, Shinjuku-ku, Tokyo, 160-8582 Japan; 5 Department of Ophthalmology, Tokyo Dental College Ichikawa General Hospital, 5-11-13 Sugano, Ichikawa-shi, Chiba, 272-8513 Japan

**Keywords:** Medical research, Medical imaging

## Abstract

The use of artificial intelligence (AI) in the diagnosis of dry eye disease (DED) remains limited due to the lack of standardized image formats and analysis models. To overcome these issues, we used the Smart Eye Camera (SEC), a video-recordable slit-lamp device, and collected videos of the anterior segment of the eye. This study aimed to evaluate the accuracy of the AI algorithm in estimating the tear film breakup time and apply this model for the diagnosis of DED according to the Asia Dry Eye Society (ADES) DED diagnostic criteria. Using the retrospectively corrected DED videos of 158 eyes from 79 patients, 22,172 frames were annotated by the DED specialist to label whether or not the frame had breakup. The AI algorithm was developed using the training dataset and machine learning. The DED criteria of the ADES was used to determine the diagnostic performance. The accuracy of tear film breakup time estimation was 0.789 (95% confidence interval (CI) 0.769–0.809), and the area under the receiver operating characteristic curve of this AI model was 0.877 (95% CI 0.861–0.893). The sensitivity and specificity of this AI model for the diagnosis of DED was 0.778 (95% CI 0.572–0.912) and 0.857 (95% CI 0.564–0.866), respectively. We successfully developed a novel AI-based diagnostic model for DED. Our diagnostic model has the potential to enable ophthalmology examination outside hospitals and clinics.

## Introduction

Dry eye disease (DED) is a leading cause of visiting an ophthalmologist. DED occurs in one out of six individuals in Japan, and the prevalence varies from 8.7 to 33.7% depending on the country and region^1–4^. Artificial intelligence (AI) by machine learning has garnered attention in the field of ophthalmology, especially in the screening and diagnosis of retinal and optic nerve disorders. These algorithms use fundus or optical coherence tomography images, which are widely used and easy to operate^5–10^. Several DED-related AI studies have been conducted in the past. Cartes et al. reported the use of machine learning-based techniques to classify the tear film osmolarity in patients with DED^11^. Maruoka et al. created a deep learning algorithm for the detection of obstructive meibomian gland dysfunction (MGD) using in vivo laser confocal microscopy^12^. da Cruz et al. created a tear film lipid layer classification algorithm based on interferometry images^13^. However, these studies evaluated only an individual DED parameter; thus, an AI algorithm for criteria-based diagnosis of DED has not been reported yet. Despite the recent innovations in retinal and optic nerve disorders, the use of AI for DED diagnosis has not progressed due to various reasons. The first reason is the absence of a simple recording device. One of the key parameters involved in the diagnosis of DED is the tear film breakup time (TFBUT), which is evaluated using slit-lamp microscopes^14^. A conventional slit-lamp microscope is non-portable, and it is difficult to determine TFBUT using video data. Yedidya et al. first reported the use of the EyeScan portable imaging system for automatic DED detection in 2007^15,16^. However, its performance did not supersede that of a conventional slit-lamp microscope. Therefore, key data, such as the TFBUT measurement video, were difficult to accumulate. The second reason is the lack of unified diagnostic criteria for DED. A recent Dry Eye Workshop (DEWS) characterized DED as a loss of homeostasis of the tear film^17^. Moreover, it emphasized the importance of global consensus in DED criteria to improve future epidemiological studies^18^. However, since DEWS did not formulate unified diagnostic criteria, the subjective and objective findings to train AI remain unknown. Therefore, to resolve these problems and challenges in developing a DED diagnostic AI, our research group invented a portable and recordable slit-lamp device named “Smart Eye Camera” (SEC). SEC is a smartphone attachment that converts the smartphone light source to cobalt blue light and magnifies × 10 to record a video of the ocular surface^19,20^. Animal and clinical studies have demonstrated sufficient function in the diagnosis of DED compared with conventional slit-lamp microscopes^19,20^. Moreover, the application of simple DED diagnosis criteria, comprising subjective symptoms and an objective TFBUT by the Asia Dry Eye Society (ADES)^21–23^, has aided in creating an AI model.

Thus, the purpose of the study was to evaluate the accuracy of the world’s first DED diagnostic AI algorithm created using ocular surface videos and the ADES DED diagnosis criteria.

## Methods

### Study design

**Figure 1:**
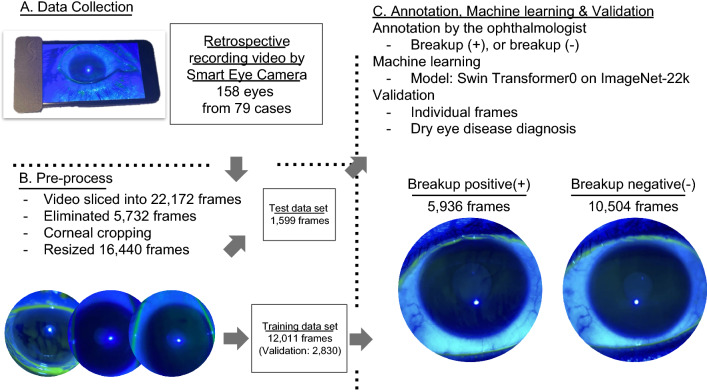
The study flowchart. (**A**) Data collection using a recordable slit-lamp device called Smart Eye Camera. Data regarding 158 eyes from 79 cases were collected. (**B**) Pre-processing before machine learning. The video was sliced into frames of 0.2-ms intervals and organized into 22,172 frames. A total of 5732 frames were eliminated as they were not focused on the ocular surface and due to noise (eyelashes, light reflex), which impeded the model from learning. The remaining 16,440 frames were resized into 384 × 384 pixels. Annotation and machine learning process. Sorting of frames: 90% of the data were assigned to the training dataset, and the remaining 10% were assigned to test dataset. A DED specialist annotated all of the frames randomly and classified them into “breakup positive(+)” or “breakup negative(-)”.

**Figure 2:**
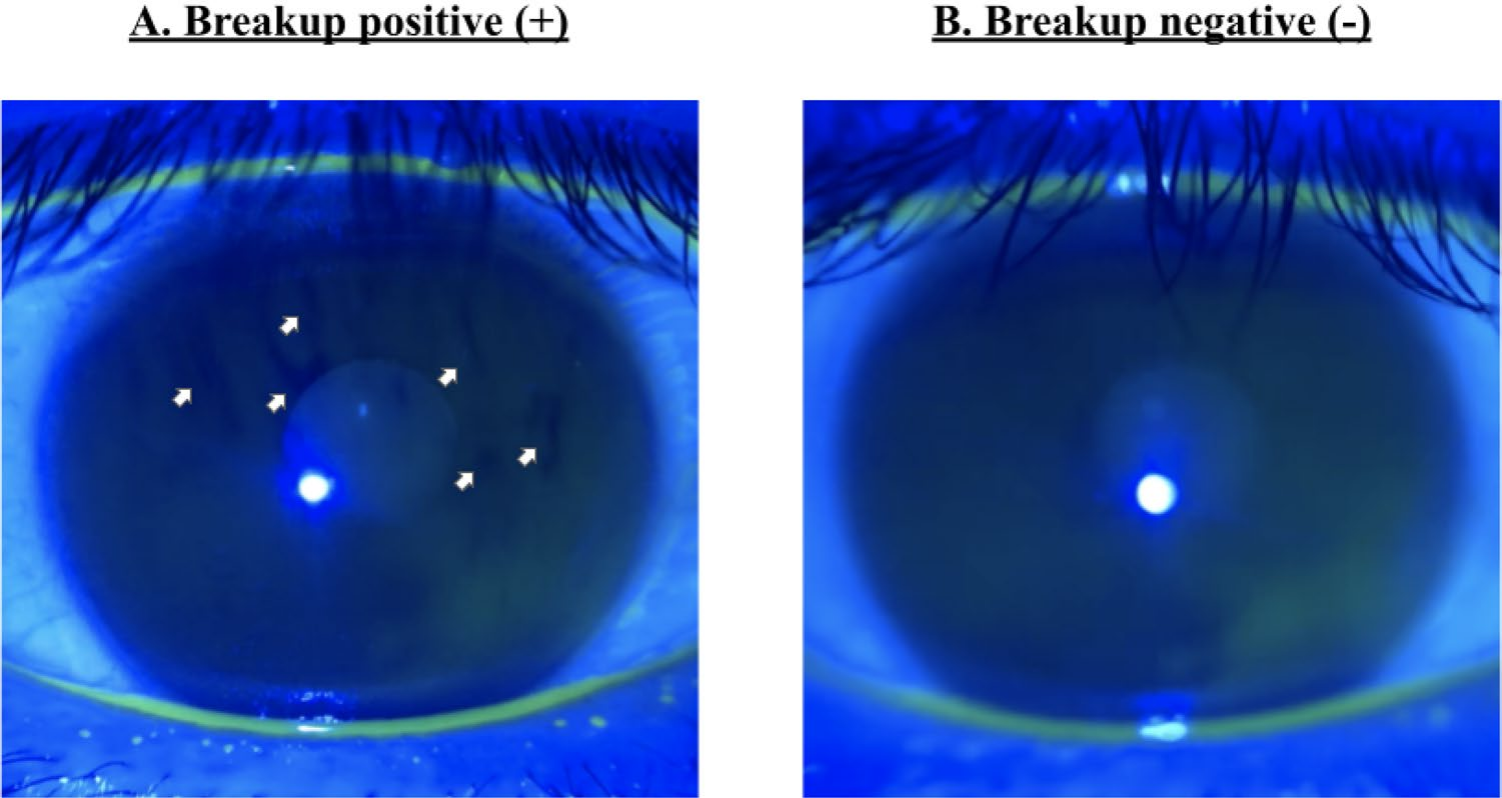
Annotation criteria. (**A**) The frames containing one or more black spots (white arrow) are classified as “breakup positive(+)”. (**B**) The frames that did not contain any black spot are classified as “breakup negative(-)”.

The ocular blue light video data were collected retrospectively from two different institutes (Keio University Hospital and Yokohama Keiai Eye Clinic). All data were collected using a mobile recording slit-light device^20^ between October 2020 and January 2021. The videos were assessed to confirm that the participant blinked at least thrice to observe the good quality of the tear film^20^. We excluded (1) images from individuals younger than 20 years of age, (2) images with severe corneal defects and/or corneal epithelitis, which makes the evaluation of the tear film difficult, and (3) images with poor quality. One case (two eyes) was excluded. Thus, videos of 158 eyes from 79 cases (all Japanese; 39 males and 40 females, 39 DED cases and 40 non-DED cases) were included in the study (Fig. 1). These videos were divided into 22,172 frames of static images. After the pre-processing in order to standardize the quality of each image and eliminate bad quality of the images (eliminate 5732 images), 90% of the data were randomly assigned to the training dataset, and the remaining 10% were assigned to the test dataset. A single DED specialist (S.S.) annotated all of the frames at random and classified them as “breakup positive (+)” or “breakup negative (−)” (Fig. 2). The machine learning process used our training dataset (details provided in the Machine learning section) to develop a machine learning model that estimated the TFBUT of each case in the validation dataset. DED diagnosis performance was calculated using the results of TFBUT estimation, and the TFBUT record in the electronic medical records (EMR) using conventional slit-lamp microscope that was considered the gold standard. DED diagnosis was defined according to the revised ADES DED criteria^21–23^.

### Mobile recording slit-light device

SEC (SLM-i07/SLM-i08SE, OUI Inc., Tokyo, Japan; 13B2X10198030101/13B2X10198030201) was used to record the ocular blue light video as a diagnostic instrument for the study. SEC is a smartphone attachment that has demonstrated sufficient diagnostic function compared with conventional slit-lamp microscopes in an animal study^20^ and several clinical studies^19,24–26^. SEC uses the same method as an ordinary slit-lamp microscope to diagnose DED using the cobalt blue light method^14^. SEC exposes the ocular surface to blue light of 488 nm wavelength emitted by the smartphone light source to enhance the fluorescence of the dye^19,20^. The fluorescence-enhanced image is recorded via the video function of the smartphone with the convex lens above the camera. An iPhone 7 or iPhone SE2 (Apple Inc., Cupertino, CA, USA) was used, with the resolution of the video set as 720 × 1280 to 1080 × 1920 pixels and the frame rate set as 30 or 60 frames per second.

### DED diagnosis

The revised ADES DED diagnostic criteria were selected as the DED diagnostic criteria^21–23^. Cases with both positive subjective symptoms and a short TFBUT (5 s or less) were considered to have DED^21–23^. The ocular surface disease index (OSDI) questionnaire was used to evaluate the subjective symptoms, and an OSDI score over 13 was defined as positive subjective symptoms^27,28^. The TFBUT measurements were obtained after administering 2.0 µl of 0.5% sodium fluorescein solution to the inferior conjunctival sac^29^. Tears were excited to visualizable green by the 488-nm wavelength blue light. TFBUT was evaluated after three blinks to confirm that fluorescein had been distributed uniformly over the whole ocular surface. TFBUT was recorded from just after each blink until the first dry spots appeared. TFBUT was measured thrice and averaged. All procedures were performed in the darkroom.

### Datasets and annotation

Seventy-nine videos containing 4434 s (average, 56.1 s) were sliced by a frame rate of 200 ms and organized into 22,172 frames. The quality exclusion criteria of eliminating incompatible frames are follows (1) the frames that did not focus on the cornea, (2) the frames which contains noise (e.g. Blurred frames, frames which do not contains cornea due to blinking, and hair or/and eyelashes in front of the cornea which may bother annotation). Thus, 5732 frames were eliminated. Next, we cropped only corneal regions to the remaining 16,440 frames in order to standardize all of the frames, then the frames were resized into 384 × 384 pixels. A single DED specialist (S.S.) annotated all images at random after the standardization of the images. The specialist annotated each image based on whether the frame was eligible for machine learning and contained the black spot on the cornea (“breakup positive [+]” or “breakup negative [−]” Fig. 2). Consequently, 5936 frames were classified as “breakup positive (+),” whereas 10,504 frames were classified as “breakup negative (−)”. After the annotating process, 90% of the data were randomly assigned to the training dataset for machine learning, whereas the remaining 10% were assigned to the test dataset for validation (training = 12,011 frames, validation = 2830 frames, test = 1599 frames, and all = 16,440 frames; training = 57 videos, validation = 14 videos, test = 8 videos, and all = 79 videos, respectively).

### Machine learning

We used Swin Transformer^30^ on ImageNet-22k^31^ as a deep learning model to develop the DED diagnostic AI. A central neural network (CNN) was used to detect the presence of breakup from the corneal image input to CNN. The training was executed with Adam as an optimizer^32^, and CossineAnnealingLR as a scheduler^33^. Moreover, augmentation [horizontal flip (0.5), transpose (0.5), and normalize with ImageNet mean/std (1)]^34^ was performed during the training to improve the accuracy. The validation dataset was divided by train vs. valid = 8 vs. 2. After the training, the output was exposed as a number from 0 to 1 to show the confidence factor. Gradient-weighted class activation mapping (GradCAM) was used for visualization^35^. The method of the TFBUT automated calculation of our model are follows (1) recognition of eye opening is define as 0 s, (2) decision of breakup positive of negative in following frames, and when the frames were judged as breakup positive (3) the model automatically calculate the time from eye opening to break up positive. The model diagnosed the case as DED when the model estimated TFBUT as 5 s or less, and the input of OSDI was over 13.

### Statistical analysis

The sample size was determined based on the feasibility of accumulating cases during the study period since this was an exploratory study and it is difficult to set the number of cases in advance to confirm the accuracy of prediction. Spearman's correlation coefficient was selected to evaluate the correlation between the TFBUT of the machine learning algorithm and the TFBUT of the EMR. To calculate the performance of the breakup estimating function for the individual frames, the accuracy (ACC), an F1-score, and an area under the curve (AUC) of the receiver operating characteristic (ROC) curve were calculated. The accuracy was calculated according to a 2 × 2 table of true positives, true negatives, false positives, and false negatives^36^. The sensitivity, specificity, positive predictive value (PPV), and negative predictive value (NPV) were calculated to define the performance of the DED diagnostic function of the machine learning model. The AUC measurement of the ROC curve was also defined to assess the more appropriate overall diagnostic function.36 Statistical analyses were performed using SPSS statistics software (ver. 25; International Business Machines Corporation, Armonk, New York, USA). Machine learning was conducted using Pytorch (Version 1.7.1.).

### Ethical approval

Our study adhered to the tenets of the Declaration of Helsinki. The trial was registered at the University hospital Medical Information Network Center (UMIN-CTR: UMIN000040321). The study protocols were approved by the Keio University School of Medicine Ethics Committee, Tokyo, Japan (IRB Number. 11000378, Approval Number. 20200021). The patient data with individual information were anonymized prior to the analysis. Patient consent was waived due to the law of the Japanese Ministry of Health, Labor and Welfare, and the IRB of Keio University School of Medicine approved a waiver or exemption for the collection of informed consent because of the retrospective study design and lack of personally identifiable information being published.

## Results

### Demographic data of the study

All of the cases are Japanese, 20–92 years of age (mean 49.85 ± 17.95). Gender difference were 39 males and 40 females with the case of 39 DED cases and 40 non-DED cases.

### Performance of the AI model in estimating the breakup of individual frames

**Figure 3:**
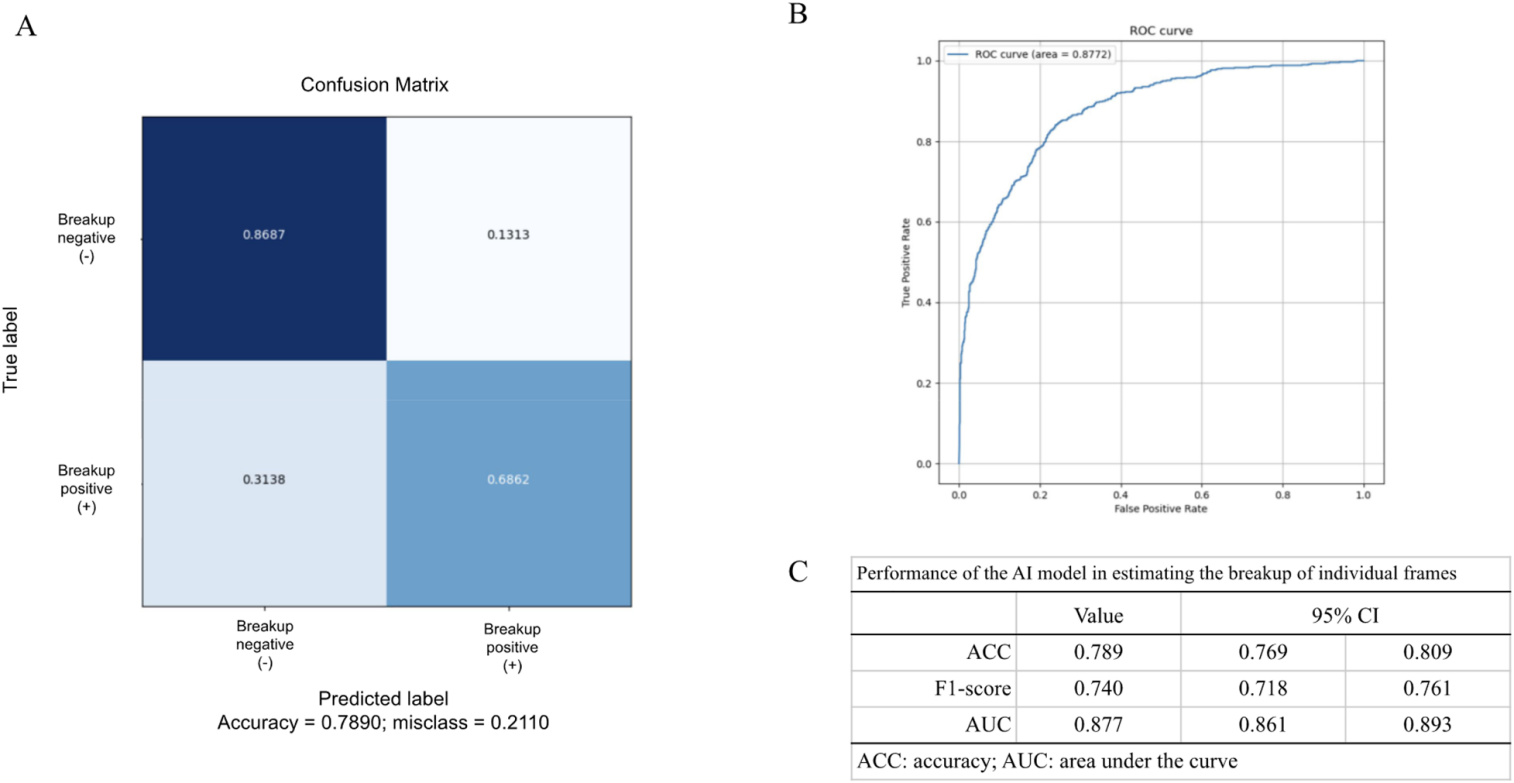
Accuracy of the trained model. (**A**) A confusion matrix of the trained model for the breakup-estimating function from individual frames. (**B**) The receiver operating characteristic curve of the trained model for the breakup-estimating function from the individual frames. (**C**) The accuracy was 0.789 (95% CI 0.769–0.809), F1-score was 0.740 (95% CI 0.718–0.761), and the area under the curve was 0.877 (95% CI 0.861–0.893).

**Figure 4:**
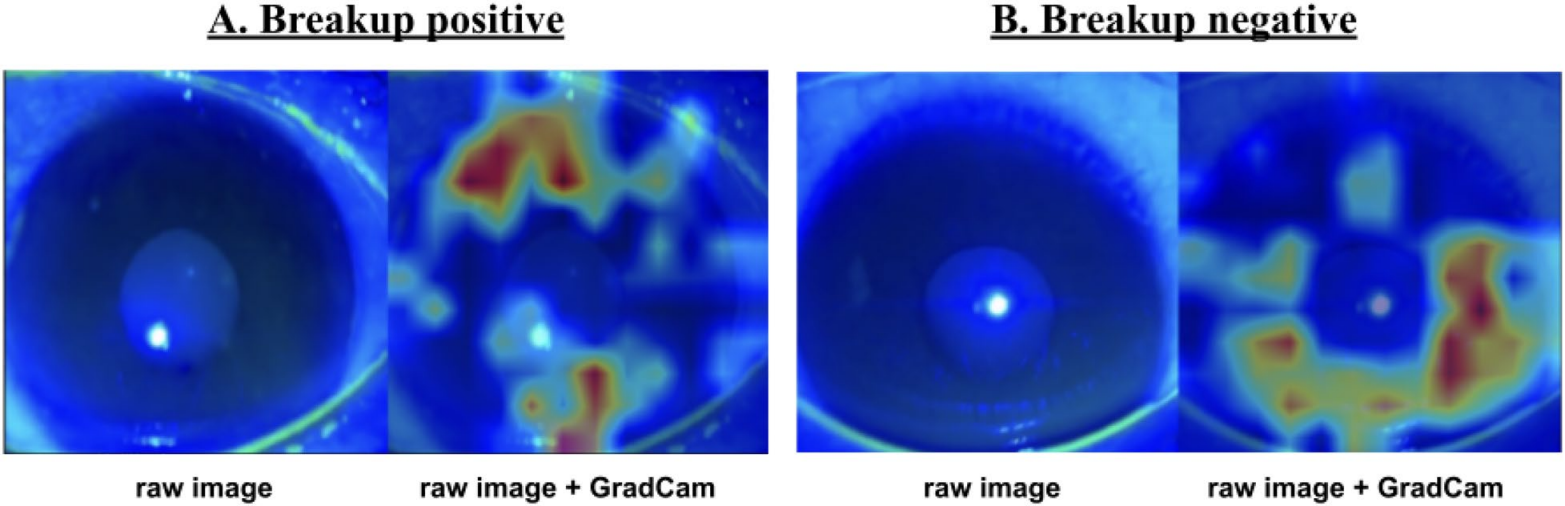
Visualization using GradCAM. (**A**) Visualization using Gradient-weighted class activation mapping (GradCAM) in a “breakup positive” frame. The raw image and GradCAM image with heatmap guidance. The black spots that display the breakup area are emphasized by the heatmap in the "raw image + GradCAM". (**B**) Visualization using GradCAM in a “breakup negative” frame. The raw image and GradCAM image with heatmap guidance. No emphasis on the cornea.

We evaluated the performance of the breakup-estimating function for individual frames. The ACC of our machine learning algorithm for individual frames was 0.789 (95% CI 0.769–0.809) (Fig. 3). The F1-score as a harmonic mean of precision and recall was 0.740 (95% CI 0.718–0.761) (Fig. 3). The AUC was 0.877 (95% CI 0.861–0.893) (Fig. 3). During visualizing with GradCAM, the breakup area was emphasized by the heatmap guide only in the “breakup positive(+)” frames, not in the “breakup negative(−)” frames (Fig. 4).

### Performance of the machine learning model in the diagnosis of DED

**Figure 5:**
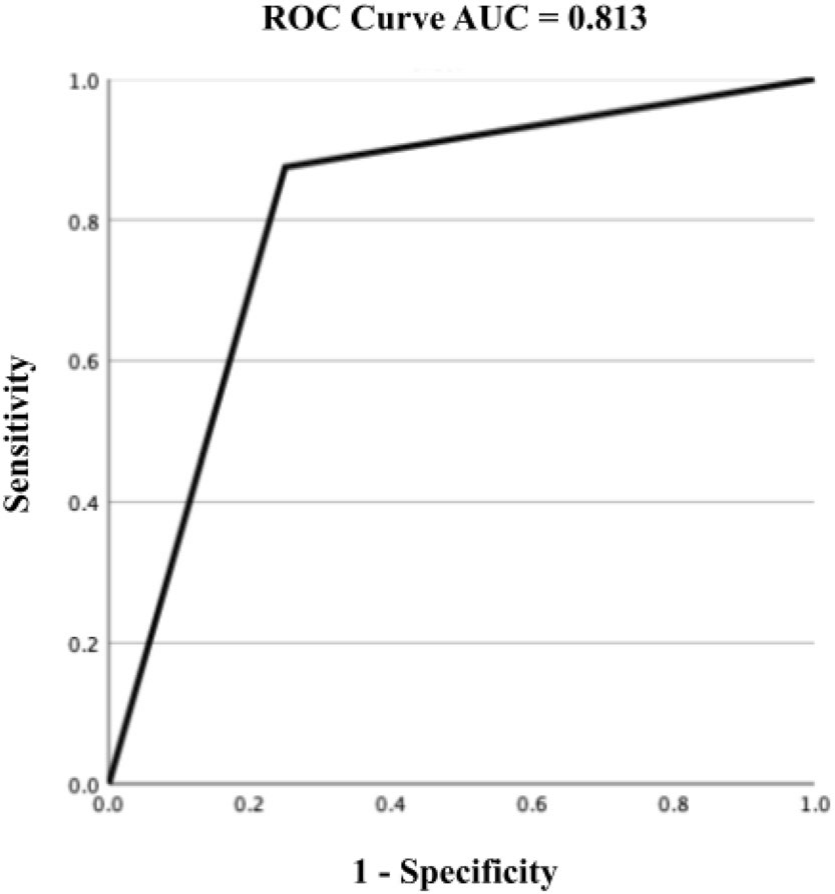
Receiver operating characteristic curve for DED diagnosis by the AI model and EMR. The area under the curve of the receiver operating characteristic curve for DED diagnosis by the AI model and EMR was 0.813 (95% CI 0.585–1.000).

The diagnostic performance for DED was assessed using TFBUT and OSDI. The sensitivity, specificity, PPV, and NPV of our model were 0.778 (95% CI 0.572–0.912), 0.857 (95% CI 0.564–0.866), 0.875 (95% CI 0.635–0.975), and 0.750 (95% CI 0.510–0.850), respectively (Table 1). Moreover, AUC was 0.813 (95% CI 0.585–1.000; Fig. 5). A moderate correlation was observed between the TFBUT determined using the machine learning algorithm and that retrieved from the EMR (r = 0.791, 95% CI 0.486–0.924). Moreover, our AI model need average 2.38 s from an input of the anterior-segment video to output of the TFBUT.

**Table 1:** Performance of the machine learning model for dye eye disease diagnosis.

	Value	95% CI	
Sensitivity	0.778	0.572	0.912
Specificity	0.857	0.564	0.866
PPV	0.875	0.635	0.975
NPV	0.750	0.510	0.850

*PPV* positive predictive value, *NPV* negative predictive value.

## Discussion

The purpose of this study was to evaluate the performance of the world’s first AI algorithm for the diagnosis of DED using ocular surface video and the ADES DED diagnosis criteria. The DED diagnostic model was developed through the estimation of TFBUT and subjective symptoms using the OSDI questionnaire.

The current model showed high ACC and AUC (ACC: 0.789, AUC: 0.877) with a moderate F1-score (F1-score: 0.740) in estimating the TFBUT validated by the individual frames. Moreover, the model demonstrated high sensitivity, specificity, and AUC (sensitivity: 0.778, specificity: 0.857, and AUC: 0.813) in DED diagnosis according to the ADES criteria^21–23^.

We considered our algorithm to be of sufficient quality based on two different point of view. (1) the interobserver reliability of TFBUT, and (2) the performance of similar imaging diagnostic models.

First, in terms of the interobserver reliability of TFBUT, Mou et al. reported that the reliability of the TFBUT in AUC was 0.695–0.792, and the correlation coefficient was 0.244–0.556 among the 147 DED and non-DED participants^37^. Moreover, Paugh et al. demonstrated an AUC of 0.917, sensitivity of 0.870, and specificity of 0.810 using the same 2.0 μl of fluorescent solution in 174 cases^38^. Our model achieved a similar function; thus, it was considered to have sufficient quality.

Second, view point from the similar imaging diagnostic AI. Ludwig et al. presented a referral identification model for diabetic retinopathy with an AUC of 0.89, F1-score of 0.85, sensitivity of 0.89, and specificity of 0.83 using 92,364 fundus camera images and fundus images taken by a smartphone^39^. Outside of the ophthalmology field, Faita et al. reported that their AI model achieved an ACC of 76.9%, AUC of 83.0%, sensitivity of 84.0%, and specificity of 70.0% in the differential diagnosis of malignant melanoma and melanocytic naevi based on the analysis of 39 ultrasonographic image samples^40^. Yang et al. reported that the coronavirus disease 2019 (COVID-19) lesion localization method, an AI model, showed an ACC of 0.884, AUC of 0.883, F1-score of 0.640, sensitivity of 0.647, specificity of 0.929, and dice coefficient of 0.575 based on the analysis of 1230 chest computed tomography scans of COVID-19 pneumonia cases^41^. These AI models are not DED diagnostic models and do not provide exact TFBUT estimation. However, these imaging methods applied a similar logic for diagnosis. Our DED diagnostic model showed a performance comparable with that of similar models.

Several reasons may explain why our model achieved high performance with a small number of samples. First, each frame was standardized before training the model. All DED video data in our study were recorded using a single type of slit-lamp device, SEC^19,20^. However, the resolution of the video and recording distance were inconsistent. Moreover, some frames contained eyelashes, reflection of the light, and other noises that were unsuitable for developing a machine-learning model. Therefore, we excluded all frames (5732 out of 22,172 frames) that failed to focus on the cornea and then standardized all frames to the same size and resolution. In a similar study, De Fauw et al. created a deep-learning diagnosis model that could be applied to multiple OCT devices and was trained using different types of OCT scans^5^. However, diversity in the resolution, contrast, and image quality of the dataset may negatively affect the machine learning process. Therefore, these pre-procedures could be one of the reasons why our model showed good performance. Second, the diagnostic method was simple. DED diagnosis was performed using only two parameters: short TFBUT and the presence of subjective symptoms. A subjective symptom, such as OSDI, was constant among the cases; therefore, our machine learning algorithm only had to define whether TFBUT was > 5 s. The recent DED diagnosis approach proposed by DEWS and ADES focused on the assessment of tear film stability^17,21^. Moreover, we applied the ADES DED criteria since our model was based on Japanese eyes. Therefore, the use of the simple ADES diagnostic criteria is another reason for the high performance. Third, we mimicked real-world examination methods. In the clinical setting, ophthalmologists use a slit-lamp microscope to evaluate objective symptoms, including TFBUT^42^. They use fluorescence solutions to stain the tear film and cobalt blue light to visualize the transparent tear layer^14^. Our model imitates these examination methods by filming the objective findings using a recordable slit-lamp device, which showed performance comparable with that of a conventional slit-lamp microscope in humans^19^. This imitation gives our model an advantage, contributing to its high performance. To improve the performance of our model, more data, especially “breakup positive” data, would have to be accumulated due to the asymmetry of the datasets (breakup positive vs. negative = 5936 vs. 10,504 frames).

Our study has several limitations.

First, the number of samples was limited. Generally, tens of thousands of patients are needed to develop a medical image-based diagnostic AI^43^. We used 16,440 frames; however, the total number of patients was 79. Moreover, numbers of DED and non-DED cases are not equal, which may be the cause of sample selection bias. Therefore, a small sample size and selection bias may have resulted in a moderate correlation coefficient for TFBUT (r = 0.791). However, we used 10% of the data to build an appropriate validation dataset^44^. Even with sufficient validation procedures and appropriate results, a larger sample with greater variability is needed to demonstrate the generalizability of the model. Furthermore, we did eliminate bad quality of images and used only good and selected images which could be causal of the bias and it need an improvement for use in the clinical setting. Hence, collecting more ocular videos is necessary to improve the accuracy and validity of the model in the future. Moreover, according to the recent perspective in DED classification, tear film-oriented diagnosis is important since the impairment of vision is closely connected with tear film instability^21^. Thus, the classification of the breakup patterns should be considered in the future.

Second, our diagnostic model was based on the updated DED diagnostic criteria defined by ADES. Thus, our model is ideal for DED diagnosis in the Asian population^21–23^. DEWS also emphasizes the importance of tear film stability, and the most frequently employed test of tear film stability is the measurement of TFBUT^17,45^. However, the prevalence of DED is reported to differ depending on the race and circumstances of the patients^1^. Hence, a validation study is needed to apply our model to different races. Moreover, our model was annotated single DED specialist so that is maybe the error of the annotation itself. Therefore, annotation by the multiple specialists are necessary in future studies.

Third, we annotated TFBUT only for the training dataset. Therefore, the TFBUT estimation is thought to be the main function of our model. To develop a more accurate DED diagnostic model, other DED findings, such as corneal fluorescein staining score^46^ and tear meniscus height^47,48^, will be required for the annotation of the fluorescence-enhanced blue light images. Moreover, additional training of MGD^49^, conjunctival lissamine green staining^50^, and conjunctival hyperemia^51^ from white diffuse illumination images will be needed to add further information to the machine learning model. Additionally, the values of the Schirmer's test^52^, tear film osmolarity^53^, and tear film lipid layer thickness interferometry^13^ may help increase the ACC of the AI algorithm. Fourth, every diagnostic process used a fluorescent solution for staining tear films. Downie LE created an instrument to measure the non-invasive tear film breakup time (NIBUT)^54^. NIBUT is measurable without any fluorescent solution; therefore, the value of the model will increase if our model can estimate NIBUT.

Despite these limitations, this study, for the first time, presents evidence for a criterion-based DED diagnostic machine-learning model. Our model demonstrated high ACC and AUC for estimating TFBUT from blue light images. Moreover, the high sensitivity, specificity, and AUC for DED diagnosis exhibited by this model might be sufficient for screening and/or providing primary medical care. All videos were recorded using a portable slit-lamp device. The availability of smartphones and teleophthalmology has increased in developed and developing countries^55^. Hence, the combination of the portable recording device and our diagnostic model has the potential to enable ophthalmology examination outside hospitals and clinics. Further studies are needed to (1) improve the accuracy of the TFBUT estimation by training with a higher number of ocular videos and classification of the breakup pattern; (2) validate the model for different races; and (3) combine additional objective findings that will be annotated by ophthalmologists.

## Data Availability

All data associated with this study can be found in the server of the Department of Ophthalmology, Keio University School of Medicine. The datasets used and/or analysed during the current study available from the corresponding author on reasonable request.

## References

[1] Alshamrani AA, Almousa AS, Almulhim AA (2017). Prevalence and risk factors of dry eye symptoms in a Saudi Arabian population. Middle East Afr. J. Ophthalmol..

[2] Lin PY, Tsai SY, Cheng CY, Liu JH, Chou P, Hsu WM (2003). Prevalence of dry eye among an elderly Chinese population in Taiwan: The Shihpai Eye Study. Ophthalmology.

[3] Hashemi H, Khabazkhoob M, Kheirkhah A (2014). Prevalence of dry eye syndrome in an adult population. Clin. Exp. Ophthalmol..

[4] Uchino M, Nishiwaki Y, Michikawa T (2011). Prevalence and risk factors of dry eye disease in Japan: Koumi study. Ophthalmology.

[5] De Fauw J, Ledsam JR, Romera-Paredes B (2018). Clinically applicable deep learning for diagnosis and referral in retinal disease. Nat. Med..

[6] Milea D, Najjar RP, Zhubo J (2020). Artificial intelligence to detect papilledema from ocular fundus photographs. N. Engl. J. Med..

[7] Yim J, Chopra R, Spitz T (2020). Predicting conversion to wet age-related macular degeneration using deep learning. Nat. Med..

[8] Ting DSW, Cheung CY, Lim G (2017). Development and validation of a deep learning system for diabetic retinopathy and related eye diseases using retinal images from multiethnic populations with diabetes. JAMA.

[9] Mitani A, Huang A, Venugopalan S (2020). Author correction: Detection of anaemia from retinal fundus images via deep learning. Nat. Biomed. Eng..

[10] Poplin R, Varadarajan AV, Blumer K (2018). Prediction of cardiovascular risk factors from retinal fundus photographs via deep learning. Nat. Biomed. Eng..

[11] Cartes C, López D, Salinas D (2019). Dry eye is matched by increased intrasubject variability in tear osmolarity as confirmed by machine learning approach. Arch. Soc. Esp. Oftalmol..

[12] Maruoka S, Tabuchi H, Nagasato D (2020). Deep neural network-based method for detecting obstructive meibomian gland dysfunction with in vivo laser confocal microscopy. Cornea.

[13] da Cruz LB, Souza JC, de Sousa JA (2020). Interferometer eye image classification for dry eye categorization using phylogenetic diversity indexes for texture analysis. Comput. Methods Programs Biomed..

[14] Gellrich M-M (2013). The Slit Lamp: Applications for Biomicroscopy and Videography.

[15] Yedidya T, Hartley R, Guillon JP, Kanagasingam Y (2007). Automatic dry eye detection. Med. Image Comput. Comput. Assist. Interv..

[16] Yedidya T, Carr P, Hartley R, Guillon JP (2009). Enforcing monotonic temporal evolution in dry eye images. Med. Image Comput. Comput. Assist. Interv..

[17] Craig JP, Nichols KK, Akpek EK (2017). TFOS DEWS II definition and classification report. Ocul. Surf..

[18] Stapleton F, Alves M, Bunya VY (2017). TFOS DEWS II epidemiology report. Ocul. Surf..

[19] Shimizu, E. *et al*. Smart eye camera: A validation study for evaluating the tear film breakup time in dry eye disease patients. *Transl. Vis. Sci. Technol.* **10**(4), 28 (2021). 10.1167/tvst.10.4.28 PMC8083120 34004005

[20] Shimizu E, Ogawa Y, Yazu H, Aketa N, Yang F, Yamane M, Sato Y, Kawakami Y, Tsubota K (2019). "Smart Eye Camera": An innovative technique to evaluate tear film breakup time in the murine dry eye disease model. PLoS One.

[21] Tsubota K, Yokoi N, Watanabe H, Members of The Asia Dry Eye Society (2020). A new perspective on dry eye classification: Proposal by the Asia Dry Eye Society. Eye 375 Contact Lens.

[22] Tsubota K, Yokoi N, Shimazaki J, Members of The Asia Dry Eye Society (2017). New perspectives on dry eye definition and diagnosis: A consensus report by the Asia Dry Eye Society. Ocul. Surf..

[23] Shimazaki J (2018). Definition and diagnostic criteria of dry eye disease: Historical 380 overview and future directions. Invest. Ophthalmol. Vis. Sci..

[24] Shimizu E, Yazu H, Aketa N, Yokoiwa R, Sato S, Yajima J, Katayama T, Sato R, Tanji M, Sato Y, Ogawa Y, Tsubota K (2021). A study validating the estimation of anterior chamber depth and iridocorneal angle with portable and non-portable slit-lamp microscopy. Sensors.

[25] Yazu H, Shimizu E, Sato S, Aketa N, Katayama T, Yokoiwa R, Sato Y, Fukagawa K, Ogawa Y, Tsubota K, Fujishima H (2021). Clinical observation of allergic conjunctival diseases with portable and recordable slit-lamp device. Diagnostics.

[26] Yazu H, Shimizu E, Okuyama S, Katahira T, Aketa N, Yokoiwa R, Sato Y, Ogawa Y, Fujishima H (2020). Evaluation of nuclear cataract with smartphone-attachable slit-lamp device. Diagnostics.

[27] Dougherty BE, Nichols JJ, Nichols KK (2011). Rasch analysis of the ocular surface 439 Disease Index (OSDI). Invest. Ophthalmol. Vis. Sci..

[28] Inomata T, Iwagami M, Nakamura M (2020). Association between dry eye and 441 depressive symptoms: Large-scale crowdsourced research using the DryEyeRhythm iPhone application. Ocul. Surf..

[29] Toda I, Tsubota K (1993). Practical double vital staining for ocular surface evaluation. Cornea.

[30] Brock, A., De, S., Smith, S. L., & Simonyan, K. High-performance large-scale image recognition without normalization. arXiv:2102.06171 (2021).

[31] Russakovsky O, Deng J, Su H (2015). ImageNet large scale visual recognition challenge. Int. J. Comput. Vis..

[32] Kingma, D. P. & Jimmy, B. Adam: A method for stochastic optimization. CoRR abs/1412.6980 (2015): n. pag.

[33] Loshchilov, I. & Hutter, F. SGDR: Stochastic gradient descent with warm restarts. arXiv: Learning (2017): n. pag

[34] Mikołajczyk, A., & Grochowski, M. Data augmentation for improving deep learning in image classification problem. In *2018 International Interdisciplinary PhD Workshop (IIPhDW), Świnouście, Poland*, 2018, pp. 117–122.

[35] Selvaraju RR, Cogswell M, Das A (2020). GradCAM: Visual explanations from deep networks via gradient-based localization. Int. J. Comput. Vis..

[36] Mander, G. T. W., & Munn, Z. Understanding diagnostic test accuracy studies and systematic reviews: A primer for medical radiation technologists [published online ahead of print, 2021 Mar 16]. J. Med. Imaging Radiat. Sci. 2021;S1939-8654(21)00037-0. 10.1016/j.jmir.2021.02.005 33741279

[37] Mou Y, Xiang H, Lin L (2021). Reliability and efficacy of maximum fluorescein tear break-up time in diagnosing dry eye disease. Sci. Rep..

[38] Paugh JR, Tse J, Nguyen T (2020). Efficacy of the fluorescein tear breakup time test in dry eye. Cornea.

[39] Ludwig CA, Perera C, Myung D (2020). Automatic identification of referral-warranted diabetic retinopathy using deep learning on mobile phone images. Transl. Vis. Sci. Technol..

[40] Faita F, Oranges T, Di Lascio N (2021). Ultra-high frequency ultrasound and machine-learning approaches for the differential diagnosis of melanocytic lesions [published online ahead of print, 2021 Mar 19]. Exp. Dermatol..

[41] Yang Z, Zhao L, Wu S, Chen YC (2021). Lung lesion localization of COVID-19 from Chest CT image: A novel weakly supervised learning method [published online ahead of print, 2021 Mar 19]. IEEE J. Biomed. Health Inform..

[42] Lemp MA, Hamill JR (1973). Factors affecting tear film breakup in normal eyes. Arch. Ophthalmol..

[43] Kusunose K (2021). Steps to use artificial intelligence in echocardiography. J. Echocardiogr..

[44] Baskin II (2018). Machine learning methods in computational toxicology. Methods Mol. Biol..

[45] Wolffsohn JS, Arita R, Chalmers R (2017). TFOS DEWS II diagnostic methodology report. Ocul. Surf..

[46] Shimizu E, Aketa N, Yazu H (2020). Corneal higher-order aberrations in eyes with chronic ocular graft-versus-host disease. Ocul. Surf..

[47] Chen Y, Li J, Wu Y, Lin X, Deng X, Yun-E Z (2021). Comparative evaluation in intense pulsed light therapy combined with or without meibomian gland expression for the treatment of meibomian gland dysfunction [published online ahead of print, 2021 Jan 18]. Curr. Eye Res..

[48] Yokoi N, Komuro A (2004). Non-invasive methods of assessing the tear film. Exp. Eye Res..

[49] Nakayama N, Kawashima M, Kaido M, Arita R, Tsubota K (2015). Analysis of meibum before and after intraductal meibomian gland probing in eyes with obstructive meibomian gland dysfunction. Cornea.

[50] Shimizu E, Ogawa Y, Saijo Y (2019). Commensal microflora in human conjunctiva; characteristics of microflora in the patients with chronic ocular graft-versus-host disease. Ocul. Surf..

[51] Yazu H, Fukagawa K, Shimizu E, Sato Y, Fujishima H (2021). Long-term outcomes of 0.1% tacrolimus eye drops in eyes with severe allergic conjunctival diseases. Allergy Asthma Clin. Immunol..

[52] Ogawa Y, Kim SK, Dana R (2013). International chronic ocular graft-vs-host-disease (GVHD) consensus Group: Proposed diagnostic criteria for 437 chronic GVHD (Part I). Sci. Rep..

[53] Tukenmez-Dikmen N, Yildiz EH, Imamoglu S, Turan-Vural E, Sevim MS (2016). Correlation of dry eye workshop dry eye severity grading system with tear meniscus measurement by optical coherence tomography and tear osmolarity. Eye Contact Lens.

[54] Downie LE (2015). Automated tear film surface quality breakup time as a novel clinical marker for tear hyperosmolarity in dry eye disease. Invest. Ophthalmol. Vis. Sci..

[55] Mohammadpour M, Heidari Z, Mirghorbani M, Hashemi H (2017). Smartphones, tele-ophthalmology, and VISION 2020. Int. J. Ophthalmol..

